# Long COVID affects working memory: assessment using a single rapid online test

**DOI:** 10.3389/fnhum.2026.1881458

**Published:** 2026-07-22

**Authors:** Aziz U. R. Asghar, Hong Kiu Yuen, Murat Aksoy, Abayomi Salawu, Heidi A. Baseler

**Affiliations:** 1 Department of Psychology, University of York, York, United Kingdom; 2 Hull York Medical School, University of Hull, Hull, United Kingdom; 3 York Biomedical Research Institute, University of York, York, United Kingdom; 4 Disability Medicine and Rehabilitation, Hull University Teaching Hospitals National Health Service (NHS) Trust, Hull, United Kingdom; 5 Hull York Medical School, University of York, York, United Kingdom

**Keywords:** cognitive dysfunction, COVID-19, long COVID, mental fatigue, post-acute COVID-19 syndrome, short-term memory

## Abstract

**Objective:**

Long COVID, or post-COVID-19 syndrome, is characterized by persistent symptoms following SARS-CoV-2 infection, including cognitive impairments such as “brain fog” that adversely affect quality of life. The aim of this study was to evaluate the impact of long COVID on working memory using a single, rapid, anonymous online survey and visual working memory quiz.

**Methods:**

We analyzed working memory scores in relation to reported long COVID status (clinically diagnosed, self-reported, and non-long COVID), age, number of COVID-19 infections, long COVID duration, and subjective ratings of brain fog severity and the overall life impact of symptoms. The study utilized the Rapid Objective Working Memory Assessment (ROWMA), a brief, gamified visual recognition task designed to maximize compliance and minimize fatigue.

**Results:**

A total of 1,064 participants aged 16 to over 85 years were recruited, with 39% reporting long COVID. Categorical regression revealed that long COVID status was the strongest predictor of working memory performance, followed by age and number of infections. Participants with long COVID performed significantly worse on the working memory quiz than non-long COVID controls, with the lowest scores observed in the clinically diagnosed group. Furthermore, working memory scores declined with age, particularly among those aged 35 and older, and decreased with multiple COVID-19 infections within the diagnosed group. Individuals in the diagnosed group reported the most severe brain fog and the greatest life impact, both of which strongly correlated with lower memory scores. Although longer long COVID duration was associated with lower memory performance, an exploratory analysis indicated this trend may have been influenced by the SARS-CoV-2 variant.

**Conclusion:**

Our results support the hypothesis that long COVID is associated with objective working memory impairment, particularly among individuals with a clinical diagnosis. These findings add to the growing body of evidence linking long COVID to objective cognitive difficulties. Moreover, the study advocates the use of rapid, single-task online cognitive assessments as a practical, scalable, and low-burden approach for evaluating working memory function in this population.

## Introduction

Long COVID, also known as post COVID-19 syndrome/condition, broadly refers to the signs and symptoms that develop during or after an infection with the SARS-CoV-2 virus that persists over a number of months and has no alternative explanation (NHS, 2021; NICE, 2020; WHO, 2022). A systematic review of long COVID prevalence, which included 194 studies with 735,006 participants, found that 45% of those who survived COVID-19 continued to experience symptoms at approximately 4 months (O’Mahoney et al., 2023).

Approximately 1.9 million people in the United Kingdom self-reported long COVID symptoms with 41% experiencing symptoms for at least 2 years and 79% reported that their symptoms negatively impacted their daily activities (Office for National Statistics, 2023); over 200 long COVID symptoms have been documented, and the most commonly reported were fatigue (72%), difficulty concentrating (51%), muscle aches (49%) and shortness of breath (48%).

Substantial cognitive performance deficits in tasks requiring memory, attention and reasoning have been reported for people with COVID-19 symptoms that persisted for up to 2 years (Bertuccelli et al., 2022; Cheetham et al., 2023; Kim et al., 2023; Millet et al., 2022; Zawilska and Kuczynska, 2022; Zhao et al., 2023; Ziauddeen et al., 2022). The cognitive impact of long COVID, often generally described as “brain fog” (including problems with memory and performing daily tasks), is a major contributor to poor functioning and reduced quality of life experienced by patients with long COVID (Jennings et al., 2022; Lam et al., 2023; Nalbandian et al., 2021; Nordvig et al., 2023). A recent systematic review and meta-analysis of studies evaluating brain fog, cognitive function and mental health symptoms in people with long COVID found that the average overall prevalence of memory loss was 21% and as high as 61% (Feltz-Cornelis et al., 2024). This considerable variation in memory loss prevalence likely reflects differences in how memory was defined, assessed or self-reported across studies.

Working memory is the process of temporarily storing and retrieving information relevant to performing the current task (Baddeley et al., 2019; Repovs and Baddeley, 2006), and is integral in everyday tasks including problem solving, holding a conversation, reading comprehension and decision making. Working memory is a core cognitive function that supports a wide range of higher-order cognitive processes, including attention, executive functioning, reasoning, learning, and comprehension (Baddeley, 2003; Diamond, 2013). Consequently, impairment of working memory may have widespread effects on everyday cognitive functioning. In line with this view, working memory deficits have been consistently reported when testing cognitive function in long COVID (Graham et al., 2021; Guo et al., 2022; Miskowiak et al., 2021, 2023). Therefore, investigating working memory provides a focused means of assessing a cognitive process that is both fundamental to higher-order cognition and commonly affected in long COVID.

The mechanisms underlying cognitive dysfunction in long COVID remain incompletely understood. However, converging evidence suggests that SARS-CoV-2 infection may have persistent effects on brain systems that support attention, executive function, and memory (Davis et al., 2023; Nalbandian et al., 2021). Working memory relies on the coordinated activity of distributed neural networks, particularly frontoparietal regions involved in the temporary storage and manipulation of information (Chai et al., 2018; Katsuki, 2012). Disruption of these networks, whether through inflammatory, vascular, or other post-viral processes, may contribute to the memory difficulties frequently reported by individuals with long COVID. The high prevalence of self-reported cognitive symptoms, together with accumulating evidence of objective cognitive deficits, highlights the need for sensitive measures capable of detecting working memory impairment in this population.

Studies investigating working memory deficits related to COVID-19 or long COVID are often measured within the context of a battery of multiple cognitive tests (Arbula et al., 2024; Cheetham et al., 2023; Cui et al., 2024; Graham et al., 2021; Guo et al., 2022; Miskowiak et al., 2021, 2023; Stavem et al., 2022). This is potentially problematic for two reasons. Firstly, when embedded within a more complex cognitive task (for example, spatial mental rotation), it is difficult to interpret performance on the working memory component alone. Moreover, it is possible that a participant’s performance on a working memory task could be affected by previous different cognitive tasks. Secondly, undertaking numerous cognitive tests within a study requires correction for multiple comparisons, which reduces statistical power. Since most people with long COVID report fatigue and difficulty concentrating, completing a lengthy survey with multiple cognitive tests would be burdensome and may decrease completion rates or yield unreliable data. Using the Rapid Objective Working Memory Assessment (ROWMA), we previously demonstrated that COVID-19 infection is associated with significantly lower working memory scores compared to no prior infection (Baseler et al., 2022).

The main aim of our study was to investigate the impact of long COVID on working memory function. To capture a broad demographic sample, we distributed an online survey with a single working memory quiz that could be completed relatively rapidly. We used the same anonymous memory quiz previously shown to detect the negative effects of COVID-19 on working memory. The quiz incorporated gamified elements to encourage greater engagement and increase completion rates (Baseler et al., 2022). In our analysis we evaluate the impact of several variables on objective memory scores including long COVID status (clinically diagnosed long COVID, self-reported long COVID and non-long COVID), age, gender, number of COVID-19 infections and long COVID duration. We also examined the relationship between objective memory scores and subjective ratings of brain fog, and overall impact of long COVID symptoms on their life.

Based on previous evidence of cognitive difficulties associated with long COVID, we hypothesized that individuals with long COVID would demonstrate poorer working memory performance than those without long COVID. We further hypothesized that poorer working memory performance would be associated with increasing age, a higher number of COVID-19 infections, and longer duration of long COVID symptoms. Finally, we hypothesized that participants reporting greater levels of brain fog and a greater overall impact of long COVID symptoms on their daily lives would exhibit lower objective memory scores.

## Materials and methods

### Ethics and participant recruitment

The study was undertaken in accordance with the Declaration of Helsinki (World Medical Association, 2013). Ethical approval was given by the Hull York Medical School Ethics Committee (Reference 22–23 41). The survey and Rapid Objective Working Memory Assessment (ROWMA) were made publicly available and data collected from 16/03/2023 to 31/12/2023. Participants with and without long COVID (aged 16+) were recruited via professional networks, social media, long COVID support groups and community advertisements. Participants provided written informed consent via a mandatory “Yes/No” prompt before starting the study. The survey and ROWMA memory quiz were fully anonymous, GDPR-compliant, and collected no personal contact details. Encrypted data was stored on the University of York’s Qualtrics platform with access restricted to the investigators. No compensation was provided.

Previous studies have inferred a diagnosis of long COVID based on ongoing symptoms (Bungenberg et al., 2022; Guo et al., 2022; Lauria et al., 2023; Thronicke et al., 2022). In our study, we adopted the NHS England definition post-COVID- syndrome (long COVID), “*…signs and symptoms that develop during or after COVID-19 and continue for more than 12 weeks and are not explained by an alternative diagnosis*” (NHS, 2021), which is broadly consistent with that of WHO (2022). To mitigate selection bias and account for healthcare barriers (e.g., lack of access or clinical validation), our methodology includes both medically diagnosed and self-reported cases as well as those without long COVID. This approach ensures a representative sample while allowing for a direct comparison between the three groups.

Participants were classified as having diagnosed long COVID if they reported receiving a diagnosis of long COVID from a healthcare professional. In the United Kingdom, long COVID is recognized as a condition in which symptoms develop during or after an infection consistent with COVID-19, persist for more than 12 weeks, and cannot be explained by an alternative diagnosis (NHS, 2021). Clinical diagnosis is typically based on an assessment of symptoms and duration, and may be supported by further investigations or referral to specialist post-COVID services where appropriate. Participants who reported persistent symptoms consistent with long COVID but had not received a formal diagnosis from a healthcare professional were classified as the self-reported long COVID group.

### Long COVID survey and memory quiz structure

**Figure 1:**
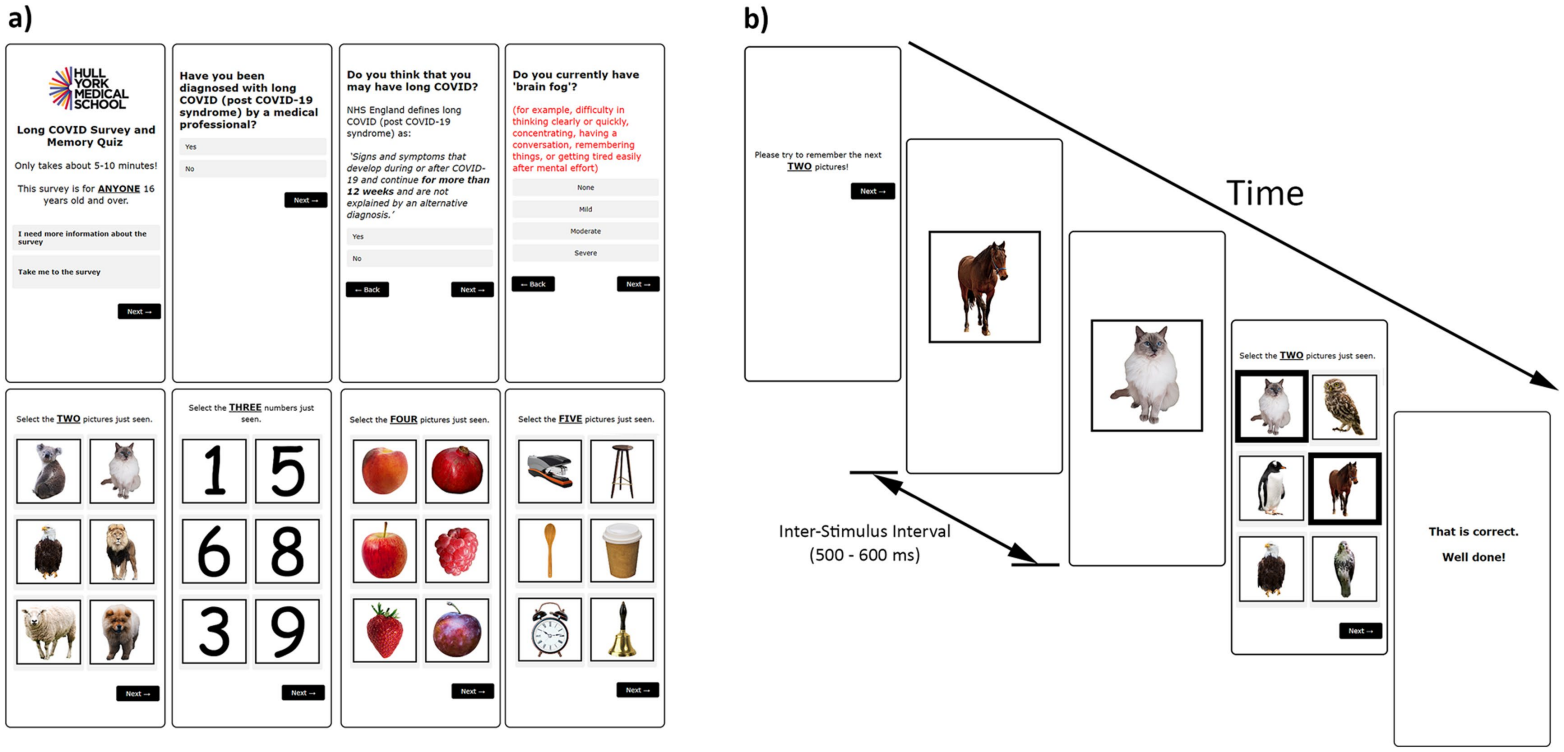
The “Long COVID Survey and Memory Quiz” presented on a smartphone. **(a)** Example questions from the survey, and examples of the animal, number, fruit and object categories shown from the memory quiz. **(b)** A series of screenshots representing a single trial from the working memory quiz.

The online assessment comprised a survey component followed immediately by an objective working memory quiz, structured similarly to our previous online evaluation paradigm (Baseler et al., 2022). Both elements were hosted via Qualtrics (University of York) and could be accessed on any internet-enabled device using a URL or QR code. Screenshots of example survey questions and a sample of the memory quiz are illustrated in Figure 1.

The survey component consisted of a series of “Yes/No” questions asking participants about their long COVID status. First, we asked, “Have you been diagnosed with long COVID (post-COVID-19 syndrome) by a medical professional?.” If a participant answered “Yes,” they were designated into the diagnosed long COVID group. Those answering “No” were then asked, “Do you think that you may have long COVID?,” and we included the NHS England definition of long COVID (post COVID-19 syndrome): “Signs and symptoms that develop during or after COVID-19 and continue for more than 12 weeks and are not explained by an alternative diagnosis” (NHS, 2021). Those that answered “Yes” to this question were designated as the self-reported long COVID group. Those who answered “No” to both questions were designated as the non-long COVID group. If they were diagnosed with long COVID or self-reported having long COVID, they were asked how many months it had been since their long COVID symptoms started (using a drop-down menu, from less than 3–60 months). We next asked long COVID participants to subjectively rate the overall life impact of their long COVID symptoms (using “None,” “Mild,” “Moderate,” or “Severe”). All participants (regardless of long COVID status) were asked whether they had been infected with COVID-19 in the last 12 weeks (“Yes/No”), and how many times they had been infected with COVID-19 (“Not had COVID-19,” “One,” “Two,” “Three,” or “Four or more times”). All participants were also asked to subjectively rate their level of “brain fog” (using “None,” “Mild,” “Moderate,” or “Severe”). The following definition of “brain fog” was provided to aid participants: “for example, difficulty in thinking clearly or quickly, concentrating, having a conversation, remembering things, or getting tired easily after mental effort.” This definition of brain fog was based on our previous findings showing that these COVID-19 cognitive symptoms were highly correlated with one another (Baseler et al., 2022). Finally, participants were asked to select their age range (16–17, 18–24, 25–34, 35–44, 45–54, 55–64, 65–74, 75–84, and 85+), gender, and country of current residence.

Following the completion of the survey, participants undertook the working memory quiz. To ensure data quality, enhance compliance, and maximize concentration, participants were instructed to complete this subsequent task in a quiet, distraction-free environment. This objective working memory assessment was developed using established principles of cognitive testing and employed a visual recognition paradigm to evaluate the short-term storage and retrieval of visual information. The task was adapted from widely used visual picture span and digit span paradigms, with stimuli presented across multiple semantic categories (Schroeder et al., 2012; Tanabe and Osaka, 2009). The construct validity of this assessment is supported by its application in a previous study using the same online visual working memory task, where it successfully discriminated between individuals with and without COVID-19 infection, demonstrated sensitivity to infection severity (hospitalized versus non-hospitalized), and significantly correlated with participants’ self-reported cognitive function and symptom severity (Baseler et al., 2022). Moreover, our working memory quiz, known as the Rapid Objective Working Memory Assessment (ROWMA), has been incorporated into a published randomized controlled pilot trial protocol approved by the Health Research Authority England (IRAS: 312024), Patient-Led Cognitive Gamified Training During Hemodialysis. In that study, ROWMA was administered alongside established cognitive assessments, including the Montreal Cognitive Assessment (MoCA) and Modified Mini-Mental State Examination (3MSE), allowing comparison with widely used measures of cognitive function (Aksoy et al., 2024).

The working memory quiz was intentionally designed to be brief and engaging to maximize completion rates and minimize participant fatigue, which is particularly important when testing individuals with long COVID who frequently report fatigue as a primary symptom. The memory task’s design prioritizes ecological validity and accessibility over the laboratory-controlled conditions typical of traditional cognitive assessments, representing a trade-off that allowed us to collect data from a larger and more diverse sample. To increase engagement, the ROWMA task employed aspects of gamification, ensuring the protocol was easy to understand through the use of familiar and engaging images. The complete stimulus set comprised 55 unique images, including 15 unique images of animals, 15 unique images of fruits, 15 unique images of objects, and 10 single-digit whole numbers ranging from 0 to 9. During the task, participants progressed through increasing difficulty levels, requiring them to recall sequences of two to five random images shown consecutively across these four semantic categories. Each stimulus image was displayed for a duration of 500 ms, with an interstimulus interval randomly varied between 500 and 600 ms. Following the sequence presentation, a recognition grid of six images was displayed, and participants were instructed to select the precise target items they had just viewed. Participant responses and overall task completion were entirely untimed and free from any time restrictions. Throughout the assessment, dynamic motivational feedback was provided following participants’ responses to maintain engagement. Upon completion of the entire quiz, participants were automatically notified of their overall performance score along with their individual scores for each of the four specific semantic sub-categories.

### Data analysis

We only included data from participants who had completed the survey and memory quiz in its entirety. Based on our inclusion criteria, participants under the age of 16 were excluded (*N* = 3). Participants that had COVID-19 infections within the last 12 weeks were also excluded from the analysis (*N* = 146), as a recent COVID-19 infection is known to affect working memory (Baseler et al., 2022). In addition, we excluded one participant who reported having long COVID for 49 months before completing the survey/memory quiz on 21st March 2023. This meant that their long-COVID symptoms would have started on the 21st of February 2019, a time before COVID-19 was identified.

A score of 1 was given for each correct trial in the memory quiz, yielding a possible maximum objective memory score of 16. Statistical Package for the Social Sciences (SPSS Version 29.0.1.0, IBM Corporation, Armonk, N. Y., United States) was utilized to perform statistical analyses. To minimize the risk of Type I errors from multiple comparisons while maintaining sensitivity to potential effects, we employed a hierarchical analytical approach. Categorical regression with optimal scaling (CATREG; SPSS) was selected because our study included a combination of nominal, ordinal, and continuous variables. CATREG uses optimal scaling to assign numerical values to categorical variables in a manner consistent with their measurement level, enabling variables measured on different scales to be incorporated into a single multivariable regression model. We used categorical regression to identify which independent variables significantly contributed to variance in memory scores. In the first categorical regression, we assessed the contributions of the following independent variables: long COVID status (three categorical/nominal groups: non-long COVID, diagnosed long COVID and self-reported long COVID), age (nine ordinal groups: 16–17, 18–24, 25–34, 35–44, 45–54, 55–64, 65–74, 75–84, and 85+), number of COVID-19 infections (five ordinal groups: none, 1, 2, 3, and 4 or more) and gender (three categorical/nominal groups: female, male, other/prefer not to say). A second categorical regression was performed using the long COVID groups only with the following independent variables: long COVID status (two groups: diagnosed long COVID and self-reported long COVID), age, number of COVID-19 infections, gender, and long COVID duration in months (ordinal, from less than 3 months up to 60 months). Next, we investigated the effect of the significant individual variables arising from the categorical regression analysis on objective memory scores, using the following non-parametric statistics, as the data were non-normally distributed: Mann–Whitney U test and Kruskal-Wallis test with Dunn’s pairwise comparisons (Bonferroni corrected). We evaluated relationships between pairs of variables using the Kendall’s tau-b correlation coefficients, as this is the most appropriate metric in correlation analyses involving ordinal variables with tied ranks. All testing was two-tailed, and we set statistical significance levels to *p-*values less than 0.05.

## Results

### Participant demographics

Participant demographics (*N* = 1,064) are detailed in Table 1. Only those who fully completed the survey and memory quiz were included. Over 94% of participants (*N* = 1,001) completed the survey and memory quiz within 15 min (median = 7.2 min): long COVID group (median = 7.3 min, *N* = 393); non-long COVID group (median = 6.9 min, *N* = 608).

**Table 1:** Participant demographics.

	All groups	Long COVID diagnosed	Long COVID self-reported	Non-long COVID
*N*	1,064 (100.0)	231 (21.7)	184 (17.3)	649 (61.0)
Age (years)				
16–17	14 (1.3)	0 (0)	2 (1.1)	12 (1.8)
18–24	128 (12.0)	7 (3.0)	18 (9.8)	103 (15.9)
25–34	184 (17.3)	28 (12.1)	34 (18.5)	122 (18.8)
35–44	223 (21.0)	53 (22.9)	50 (27.2)	120 (18.5)
45–54	262 (24.6)	78 (33.8)	38 (20.7)	146 (22.5)
55–64	188 (17.7)	54 (23.4)	36 (19.6)	98 (15.1)
65–74	49 (4.6)	8 (3.5)	6 (3.3)	35 (5.4)
75–84	15 (1.4)	3 (1.3)	0 (0)	12 (1.8)
85+	1 (<1.0)	0 (0)	0 (0)	1 (0.2)
Gender				
Female	806 (75.8)	203 (87.9)	138 (75.0)	465 (71.6)
Male	244 (22.9)	27 (11.7)	40 (21.7)	177 (27.3)
Other	14 (1.3)	1.0 (0.4)	6 (3.3)	7 (1.1)
Countries				
UK	883 (83.0)	174 (75.3)	150 (81.5)	559 (86.1)
Non-UK	181 (17.0)	57 (24.7)	34 (18.5)	90 (13.9)
Number of COVID infections				
0	181 (17.0)	1 (0.4)	5 (2.7)	175 (27.0)
1	525 (49.3)	115 (49.8)	93 (50.5)	317 (48.8)
2	278 (26.1)	84 (36.4)	62 (33.7)	132 (20.3)
3	67 (6.3)	24 (10.4)	22 (12.0)	21 (3.2)
4+	13 (1.2)	7 (3.0)	2 (1.1)	4 (0.6)

Data values are presented as sample size (%).

### Categorical regression and objective memory scores

A categorical regression revealed that significant effects were found, in order of importance, for long COVID status, age and number of COVID-19 infections (Table 2). Gender did not have a significant effect on memory scores. A second categorical regression was performed for the two long COVID groups only to assess the additional contribution of long COVID duration on memory scores. Table 3 shows that the significant factors were, in order of importance: age, long COVID duration, and number of COVID-19 infections. Long COVID subgroup or gender did not have a significant effect on memory scores. We then went on to evaluate each of the significant factors independently. We used non-parametric statistics in all these subsequent analyses as a Kolmogorov–Smirnov test revealed that objective memory scores were not normally distributed [*D*(1,064) = 0.26, *p* < 0.001].

**Table 2:** Categorical regression analysis and objective memory scores for non-long COVID, diagnosed long COVID and self-reported long COVID participants.

Adjusted regression coefficient *R*^2^ = 0.114, Fisher’s *F*-test = 13.479, ***p < 0.*001**				
Independent variables	Beta	*F*	*p*	Importance (pratt)
Long COVID status (non-long COVID, diagnosed long COVID, self-reported long COVID groups)	0.240	60.221	**<0.001**	0.554
Age	−0.184	48.074	**<0.001**	0.315
Number of COVID-19 infections	−0.110	7.686	**<0.001**	0.133
Gender	0.011	0.402	0.669	−0.002

Significant *p*-values are given in bold.

**Table 3:** Categorical regression analysis and objective memory scores for diagnosed long COVID and self-reported long COVID participants only.

Adjusted regression coefficient *R*^2^ = 0.079 Fisher’s *F*-test = 3.207, ***p < 0.*001**				
Independent variables	Beta	*F*	*p*	Importance (pratt)
Age	−0.238	32.297	**<0.001**	0.529
Long COVID duration	−0.145	6.491	**<0.001**	0.260
Number of COVID infections	−0.139	4.678	**0.003**	0.155
Long COVID subgroup (diagnosed long COVID, self-reported long COVID groups)	0.044	1.360	0.244	0.055
Gender	0.007	0.044	0.957	0.001

Significant *p*-values are given in bold.

### Effect of long COVID status on working memory

**Figure 2:**
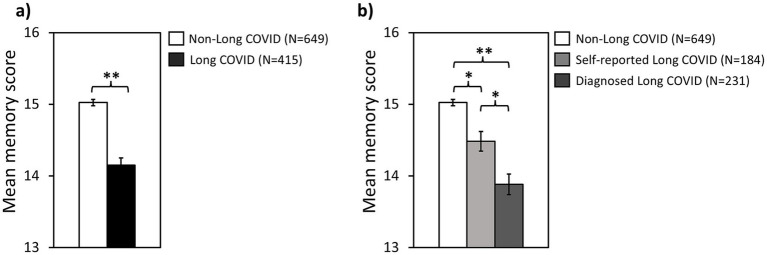
The effect of long COVID status on objective memory scores. **(a)** Non-long COVID group compared to both long COVID groups combined (diagnosed plus self-reported long COVID), and **(b)** non-long COVID group compared to self-reported and diagnosed long COVID groups separately. Error bars represent standard error of the mean. *p < 0.01, **p < 0.001.

Figure 2a shows that the objective memory scores are significantly reduced in the two long COVID groups combined [Mean (M) ± standard error of the mean = 14.15 ± 0.10] compared to the non-long COVID group (*M* = 15.02 ± 0.05), Mann–Whitney *U* = 167938.50, *z* = −7.12, *p <* 0.001, *r* = 0.22. Next, we divided the long COVID group into subgroups: those who suspected they may have long COVID (self-reported), and those who were diagnosed with long COVID by a medical professional. Figure 2b shows that objective memory scores were highest in the non-long COVID group but were significantly reduced in each of the long COVID subgroups, with the lowest scores in the diagnosed group. A Kruskal-Wallis test revealed a significant difference between the mean memory scores across groups, *H*(2) = 61.38, *p <* 0.001, η^2^ = 0.06. Dunn’s pairwise comparison tests with Bonferroni correction revealed significant differences between the non-long COVID group (*M* = 15.02 ± 0.05) and the self-reported long COVID (*M* = 14.48 ± 0.14), *z* = 3.20, *p* = 0.0042, *r* = 0.11, and between the non-long COVID and diagnosed long COVID groups (*M* = 13.88 ± 0.15), *z* = −7.71, *p <* 0.001, *r* = 0.26. In addition, there was a significant difference between the self-reported and diagnosed long COVID groups, *z* = −3.28, *p =* 0.0031, *r* = 0.16.

### Effect of age on working memory

**Figure 3:**
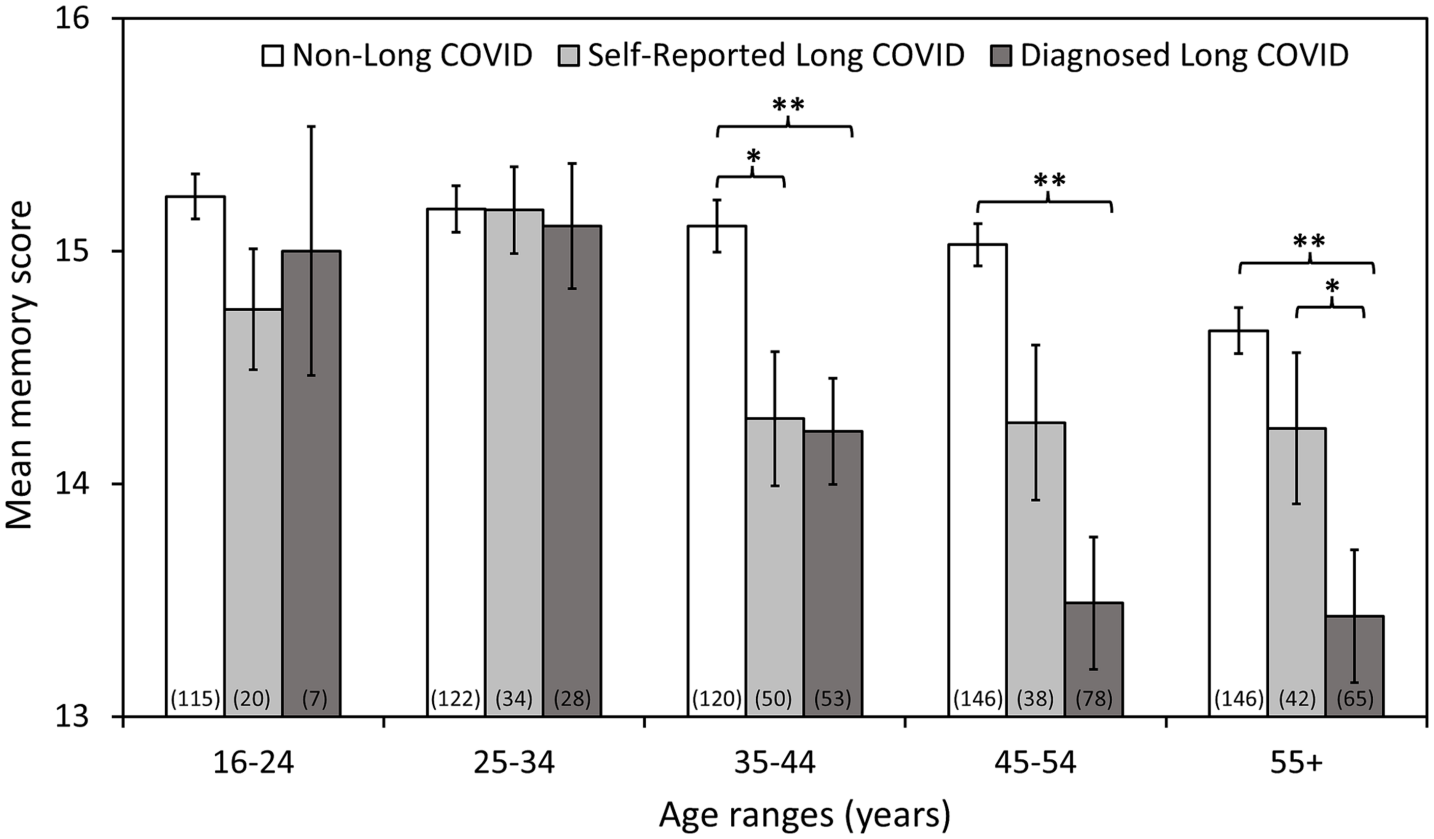
The effect of age on objective memory scores in the non-long COVID, self-reported long COVID and diagnosed long COVID groups. The sample size is given in brackets for each data bar. Error bars represent the standard error of the mean. Asterisks indicate significant differences between groups for each age range category. *p < 0.05, **p < 0.001.

We found that objective memory scores decreased significantly with age for both the non-long COVID and the diagnosed long COVID groups but not for the self-reported long COVID group. A Kendall’s tau-b correlation showed a negative relationship between mean memory score and age for both the non-long COVID (τb = −0.155, *p <* 0.001) and diagnosed long COVID (τb = −0.208, *p <* 0.001) groups across the nine age categories. The self-reported long COVID group did not have a significant relationship between age and mean memory scores (τb = −0.099, *p =* 0.098). Participants aged 16–17 years old were combined with those aged 18–24 years old, and participants above 55 years old were combined to increase category sample size (see Table 1). Figure 3 shows the effect of age on memory scores for all three groups for the five age categories. A Kruskal-Wallis test was performed at each of the five age categories to determine differences between groups based on long COVID status. There was no significant effect of long COVID status on memory scores for the 16–24 year-old age category [*H*(2) = 4.201, *p =* 0.122, η^2^ = 0.02], or the 25–34 year-old age category [*H*(2) = 0.004, *p =* 0.998, η^2^ = 0]. However, there was a significant effect of long COVID status on memory scores for the 35–44 [*H*(2) = 18.539, *p <* 0.001, η^2^ = 0.08], 45–54 [*H*(2) = 24.377, *p <* 0.001, η^2^ = 0.09] and 55 + year-old categories [*H*(2) = 15.416, *p <* 0.001, η^2^ = 0.05]. Dunn’s pairwise comparisons (with Bonferroni correction for multiple tests) revealed that the memory scores in the diagnosed long COVID group were significantly reduced compared to the non-long COVID group (all *p* < 0.001) in the 35–44 (*z* = −4.01; *r* = 0.30), 45–54 (*z* = −4.88; *r* = 0.33), and 55 + year-old categories (*z* = −3.88; *r* = 0.27). The memory scores for the self-reported long COVID group were significantly reduced compared to the non-long COVID group in the 35–44 age category (*z* = −2.70; *r* = 0.21; *p =* 0.021). In the 55 + age category, the memory scores for the diagnosed long COVID group were significantly smaller than for the self-reported group (*z* = −2.53; *r* = 0.24; *p =* 0.034).

### Effect of the number of COVID-19 infections on working memory

**Figure 4:**
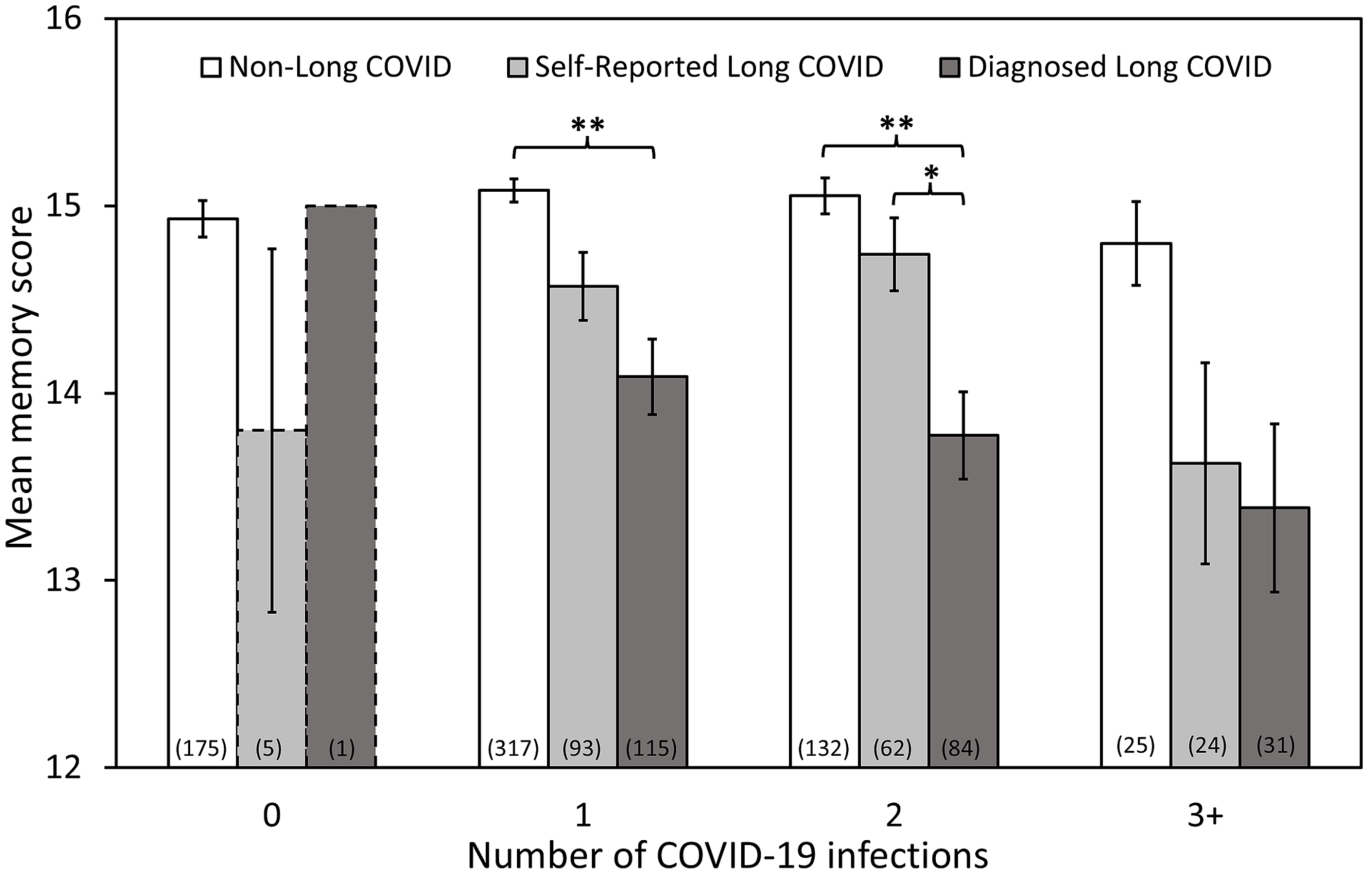
The effect of COVID-19 infections on objective memory scores in the non-long COVID, self-reported long COVID and diagnosed long COVID groups. The sample size is given in brackets for each data bar. Error bars represent the standard error of the mean. Asterisks indicate significant differences between groups for the number of COVID-19 infections. The dashed border on vertical bars (zero COVID-19 infections category) indicates the small number of participants who stated having long COVID despite an apparent absence of COVID-19 infection. *p < 0.01, **p < 0.001.

Objective memory scores decreased significantly with increasing number of COVID-19 infections for the diagnosed long COVID group (τb = −0.113, *p =* 0.043). However, there was no significant correlation between the number of infections and memory scores for either the self-reported long COVID group (τb = −0.040, *p =* 0.527) or the non-long COVID group (τb = 0.001, *p =* 0.970). Next, we compared the impact of the number of COVID-19 infections on memory scores between the non-long COVID, self-reported and diagnosed long COVID groups. Since there were only 13 participants with four or more COVID-19 infections we combined these participants with those who had three infections (*N* = 67). Figure 4 shows the effect of the number of COVID-19 infections on memory scores for all three long COVID status groups for the four infection categories. A Kruskal-Wallis test was performed at each of the four infection categories to determine differences between groups based on long COVID status. When participants had one or two COVID-19 infections more than 12 weeks prior to completing the survey and memory quiz, there was a significant effect of long COVID status on memory scores [one COVID-19 infection: *H*(2) = 23.046, *p <* 0.001, η^2^ = 0.04; two COVID-19 infections: *H*(2) = 25.352, *p <* 0.001, η^2^ = 0.09]. There was no significant effect of long COVID status on memory scores in participants with 3 or more infections [*H*(2) = 5.228, *p =* 0.073, η^2^ = 0.04]. Dunn’s pairwise comparisons (with Bonferroni correction for multiple tests) revealed that the memory scores in the diagnosed long COVID group were significantly reduced compared to the non-long COVID group (*p <* 0.001) in the one (*z* = −4.70; *r* = 0.23) and two COVID-19 infection categories (*z* = −5.00; *r* = 0.34). The memory scores for the diagnosed long COVID group were significantly reduced compared to the self-reported group in the two-infection category (*z* = −3.05; *r* = 0.25, *p =* 0.007). For the zero-infection category, there was an insufficient number of participants to perform statistical analysis (these participants stated having long COVID despite an apparent absence of COVID-19 infection).

### Effect of long COVID duration on working memory

**Figure 5:**
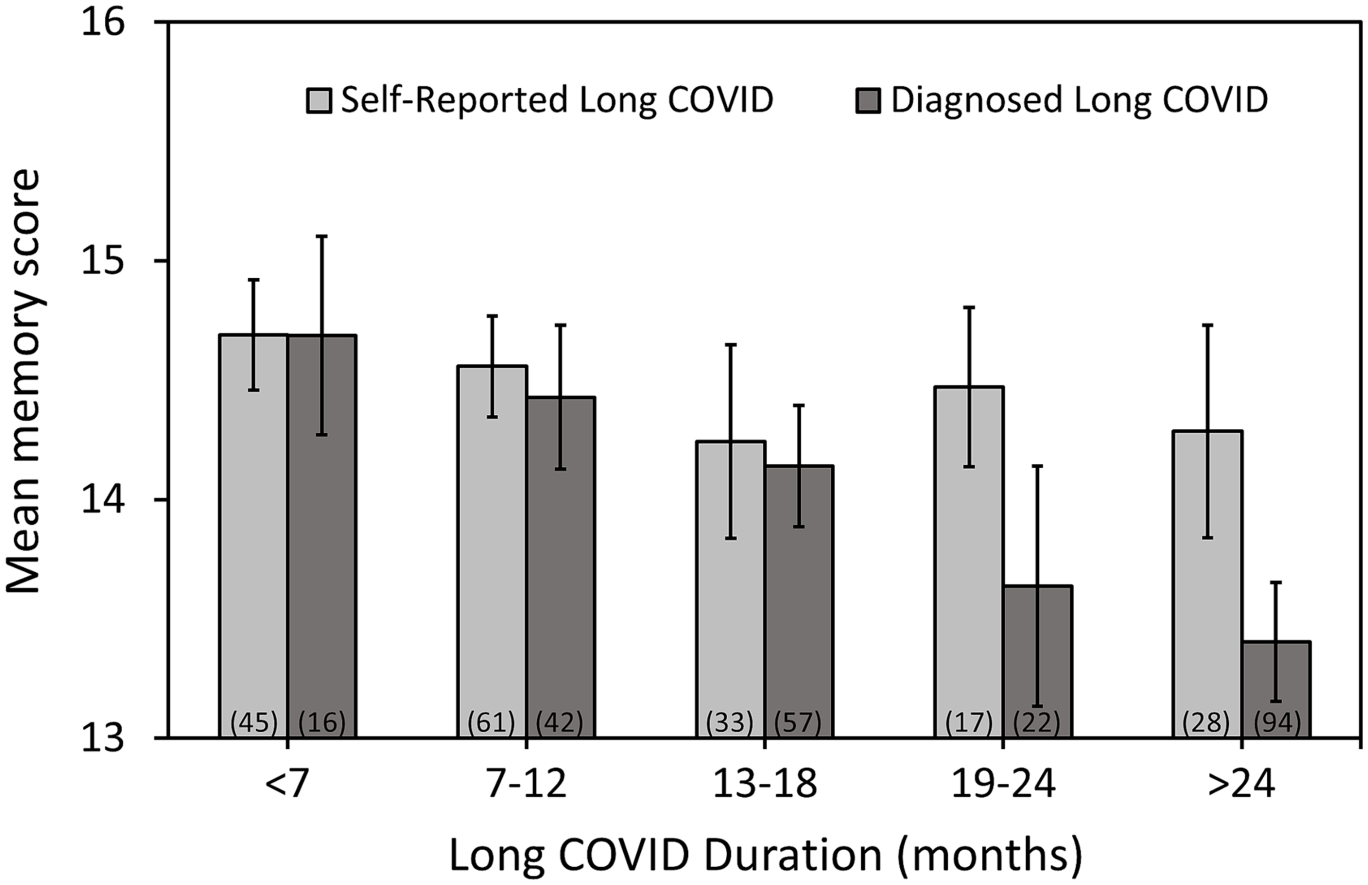
The effect of long COVID duration on objective memory scores in the self-reported and diagnosed long COVID groups. The sample size is given in brackets for each data bar. Each bar represents a different sample of participants (between-subject design). Error bars represent the standard error of the mean.

As the number of months of having long COVID increased, the memory scores decreased significantly in the diagnosed long COVID group (τb = −0.178, *p <* 0.001) but not in the self-reported long COVID group (τb = −0.021, *p =* 0.712). Next, we compared the impact of long COVID-19 duration on memory scores between the self-reported long COVID and diagnosed long COVID groups. We combined participants into six-month long COVID-19 duration categories. Figure 5 shows the effect of long COVID duration on memory scores for the self-reported long COVID and diagnosed long COVID groups. A Mann–Whitney U test found no significant difference between the self-reported long COVID and diagnosed long COVID groups for any of the five long COVID duration categories, Bonferroni-corrected for five duration categories.

### COVID-19 variants and working memory

The SARS-CoV-2 virus evolved into different variants throughout the pandemic, meaning participants likely contracted different strains depending on when they were infected. Research indicates that those infected during earlier variant waves face a higher probability of persistent long COVID symptoms, including cognitive and memory impairments (Diexer et al., 2023). Hence, lower memory scores associated with longer COVID duration (Figure 5) may have been influenced by the specific SARS-CoV-2 variant. In a post-hoc analysis, participants based in the United Kingdom were grouped by the variant dominant at the onset of their long COVID symptoms. Adopting the methodology employed in major community-based epidemiological trials, the REal-time Assessment of Community Transmission (REACT) studies, we used the date of infection as a proxy for the dominant circulating strain to classify variant cohorts without requiring universal viral sequencing, which is often impractical and resource-intensive in large population-based studies (Atchison et al., 2023; Elliott et al., 2022). This onset was estimated by subtracting the reported symptom duration (months) from the date the survey and quiz were completed. Our variant groups were based on the time periods used previously for the United Kingdom: (1) wild-type, prior to December 2020; (2) alpha, from the beginning of December 2020 to the end of April 2021; (3) delta, from the beginning of May 2021 to the end of December 2021; (4) omicron, from the beginning of January 2022 onwards (Atchison et al., 2023; Elliott et al., 2022).

**Figure 6:**
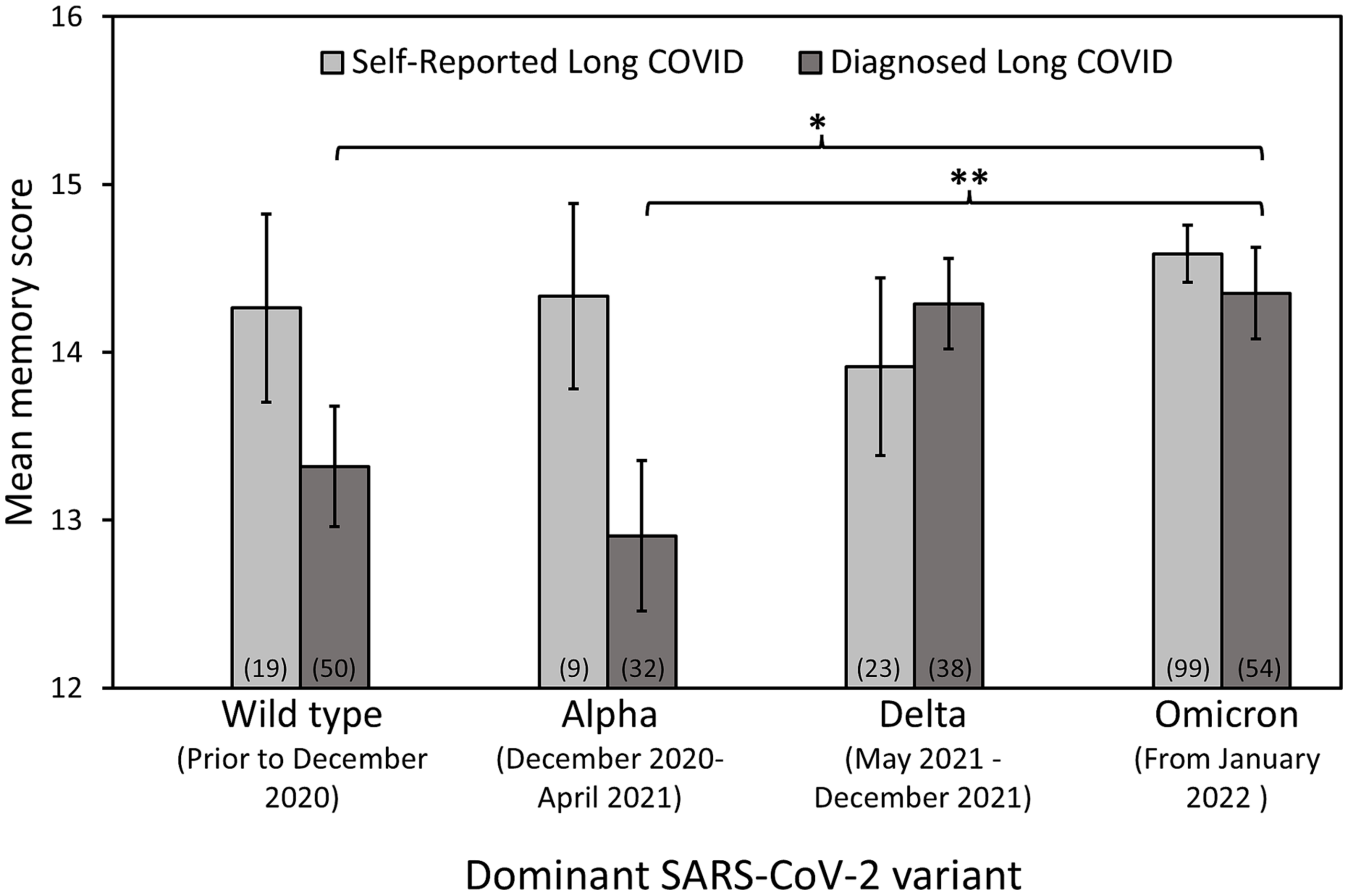
The dominant SARS-CoV-2 variant and objective memory scores in the self-reported and diagnosed long COVID groups in participants only from the United Kingdom. The long COVID participants were divided into four groups (Wild type, Alpha, Delta and Omicron) according to the dominant variant at the time when their long COVID symptoms began. The sample size is given in brackets for each data bar. Error bars represent the standard error of the mean. *p < 0.05, **p < 0.01.

A Kendall’s tau-b correlation showed a significant relationship between the SARS-CoV-2 variant that was dominant at the time at which long COVID symptoms started and the duration of long COVID symptoms for both the self-reported (τb = −0.740, *p <* 0.001) and diagnosed (τb = −0.869, *p <* 0.001) long COVID groups. Figure 6 illustrates the objective memory scores for each dominant SARS-CoV-2 variant in the self-reported and diagnosed long COVID groups in United Kingdom participants only. For participants with diagnosed long COVID, there was a significant difference in mean memory scores across the four SARS-CoV-2 variants [Kruskal-Wallis test, *H*(3) = 14.140, *p =* 0.003, η^2^ = 0.07]. For participants with diagnosed long COVID, we found significantly lower mean memory scores for the wild type compared to the omicron variant (*z* = 2.78; *r* = 0.27, *p =* 0.033) and alpha compared to the omicron variant (*z* = −3.17; *r* = 0.34, *p =* 0.009). There was no significant difference in mean memory scores across the four SARS-CoV-2 variants for participants with self-reported long COVID [Kruskal-Wallis test, *H*(3) = 1.236, *p =* 0.744, η^2^ = 0.01].

### Relationship between “brain fog,” “impact of long COVID symptoms on life” and objective memory scores

**Figure 7:**
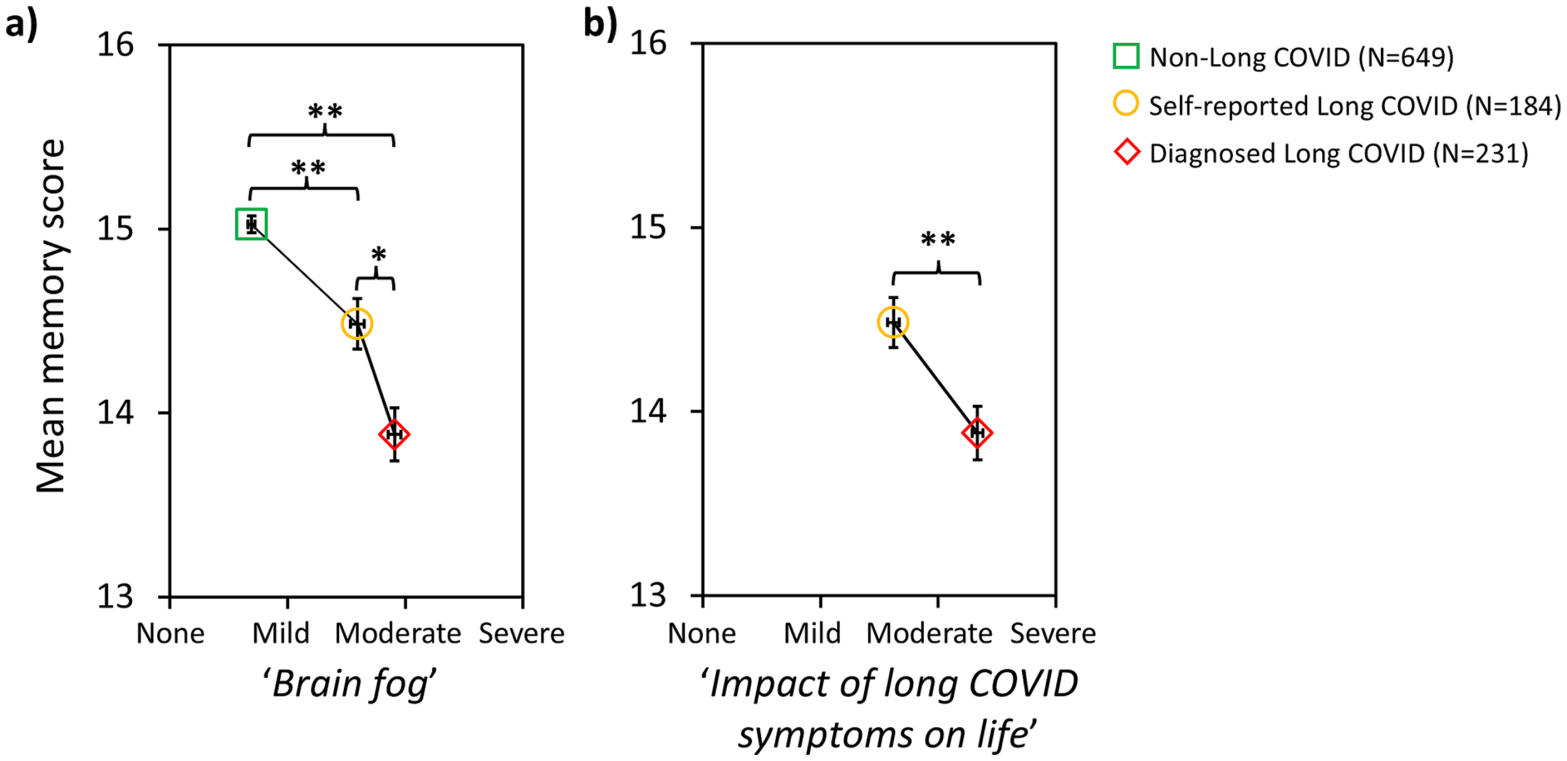
Subjective ratings of **(a)** “Brain fog” and **(b)** “Impact of long COVID symptoms on life” versus memory scores. The vertical error bars represent the standard error of the mean for memory scores, and horizontal error bars indicate the standard error of the mean for “*Brain fog*” or “*Impact of long COVID symptoms on life*” ratings. Statistical significance values refer to group differences in ratings for “*Brain fog*,” and for “*Impact of long COVID symptoms on life*” *p < 0.01, **p < 0.001.

There was a significant negative correlation between the subjective ratings for “*Brain fog*” and objective memory scores across all participants (τb = −0.185, *p <* 0.001). Figure 7a shows the mean subjective ratings for “*Brain fog*” versus objective memory scores for each of the non-long COVID, self-reported long COVID and diagnosed long COVID groups. A Kruskal-Wallis test revealed a significant relationship between long COVID status and “*Brain fog*” ratings [*H*(2) = 331.90, *p <* 0.001, η^2^ = 0.31]. Dunn’s pairwise comparisons (with Bonferroni correction for multiple tests) revealed that compared to the non-long COVID group, subjective ratings of “*Brain fog*” were significantly more severe in the self-reported (*z* = −11.61; *r* = 0.40, *p <* 0.001) and diagnosed long COVID groups (*z* = 16.42; *r* = 0.55, *p* < 0.001). Moreover, the subjective ratings of “*Brain fog*” in the diagnosed long COVID group were significantly more severe compared to the self-reported long COVID group (*z* = 2.92; *r* = 0.14, *p* = 0.011). There was a significant negative correlation between the subjective ratings for “*Impact of long COVID symptoms on life*” and objective memory scores across all participants with long COVID (τb = −0.169, *p <* 0.001). Figure 7b shows the mean subjective ratings of the “*Impact of long COVID symptoms on life*” versus objective memory scores for the self-reported and diagnosed long COVID groups. The subjective ratings of “*Impact of long COVID symptoms on life*” in the diagnosed long COVID group were significantly worse compared to the self-reported long COVID group (Mann–Whitney U test = 10372.00, *z* = −9.61, *p <* 0.001, *r* = 0.47). There was a significant correlation between the subjective ratings for “*Brain fog*” and “*Impact of long COVID symptoms on life*” in the long COVID (self-reported and diagnosed) group (τb = 0.405, *p <* 0.001).

## Discussion

Our study supports the hypothesis that long COVID may affect working memory. Long COVID status (diagnosed or self-reported) was the strongest predictor among the variables examined, followed by age and infection frequency. Using the same rapid online test as Baseler et al. (2022), we confirmed that both long COVID groups performed significantly worse than non-long COVID controls. These results align with previous studies reporting memory deficits in individuals with continuing COVID-19 symptoms (Bertuccelli et al., 2022; Graham et al., 2021; Guo et al., 2022; Miskowiak et al., 2021, 2023; Zhao et al., 2023). Our diagnosed long COVID group’s mean score (13.88) was lower than non-hospitalized COVID-19 patients (14.73) and more comparable to hospitalized patients (13.46) from our previous work (Baseler et al., 2022), suggesting poorer working memory performance in this population.

Our results link working memory deficits to “brain fog,” a hallmark of long COVID (Aghajani Mir, 2023; Kalak et al., 2022; Millet et al., 2022; Premraj et al., 2022). Objective memory scores negatively correlated with brain fog severity, which was highest in the diagnosed group, followed by self-reported and non-long COVID groups. This finding aligns with the significantly higher rates of memory impairment observed in long COVID participants reporting brain fog (Jennings et al., 2022).

The diagnosed long COVID group reported a more severe impact on daily life with lower memory scores than the self-reported group. These findings suggest that working memory deficits contribute to the overall functional burden of the condition, a link further supported by the strong correlation between memory scores, subjective brain fog and the reported impact of symptoms on life. This aligns with previous studies showing that long COVID can significantly reduce quality of life, including persistent physical symptoms, mental health problems, ability to cope, reduced functional status and employment issues (Aiyegbusi et al., 2021; Graham et al., 2021; Kennelly et al., 2023; Kim et al., 2023; Natarajan et al., 2023; Ziauddeen et al., 2022).

Previous studies on cognitive function in long COVID have typically classified participants into a long COVID group based on persistent symptomology rather than clinical diagnosis by a medical professional (Bungenberg et al., 2022; Guo et al., 2022; Lauria et al., 2023; Thronicke et al., 2022). One of the strengths of our study is that we included a group of long COVID participants who were clinically diagnosed. Medical professionals will diagnose patients with long COVID based on multiple sources of evidence, including case history, signs and symptoms, physical assessments/clinical tests, and may be supported by members of a multi-disciplinary team. In our study, we added a separate self-reported long COVID group to represent those who may have long COVID but were not clinically diagnosed.

Objective memory scores in the non-long COVID group decreased significantly with age, consistent with our previous study (Baseler et al., 2022) and others (Brockmole and Logie, 2013; Park et al., 2002). Notably, this age-related decline was more pronounced in the diagnosed long COVID group. While memory scores were comparable between diagnosed and control groups under age 35, working memory scores were significantly lower in the diagnosed long COVID group for those aged 35 and above. Our finding that long COVID may be an additional factor contributing to memory impairment in older adults aligns with broader evidence that aging increases vulnerability to post-COVID-19 cognitive deficits (Baseler et al., 2022; Blomberg et al., 2021; Corbett et al., 2023; Cui et al., 2024; Finamore et al., 2024; Nalbandian et al., 2021; Stavem et al., 2022).

SARS-CoV-2 reinfection increases the risk of persistent neurological symptoms (Bowe et al., 2022). Consistently, we found that memory scores decreased as the number of infections increased, specifically within the diagnosed long COVID group. This supports the view that reinfection exacerbates cognitive dysfunction in these individuals. A small number of participants (*N* = 6) reported long COVID despite no known infection. This could be due to a lack of initial testing, asymptomatic infection (Gao et al., 2021), vaccine side effects (Finterer and Scorza, 2022), or reporting errors.

In our cross-sectional study, memory scores decreased as long COVID duration increased. However, this association may be influenced by differences in the SARS-CoV-2 variants circulating at the time participants developed long COVID symptoms. Previous studies have reported that earlier variants may be associated with a greater risk of persistent cognitive symptoms than later variants (Hampshire et al., 2024). In a post-hoc exploratory analysis, we divided participants from the United Kingdom with long COVID into four variant groups (wild-type, alpha, delta and omicron) based on the dominant SARS-CoV-2 strain circulating during the estimated onset of their long COVID symptoms (Atchison et al., 2023; Elliott et al., 2022). Participants in the diagnosed long COVID group whose symptoms were reported to have begun during periods dominated by the wild-type and alpha variants had lower memory scores than those assigned to the omicron period. However, because variant classification was based on estimated periods of variant predominance rather than confirmed sequencing data, these findings should be interpreted with caution and considered hypothesis-generating. Future studies using confirmed infection dates and variant identification are needed to determine the extent to which SARS-CoV-2 variants contribute to long-term cognitive outcomes.

Long COVID status was based on participant self-report rather than independent clinical verification, and some degree of misclassification cannot be excluded. Nevertheless, the diagnosed long COVID group had lower memory scores than the self-reported group, suggesting that working memory deficits were more severe in those diagnosed by a medical professional. Furthermore, memory scores in the self-reported long COVID group showed no significant decline associated with age, infection frequency, long COVID duration, or probable viral variant. Participants with more severe symptoms, such as brain fog, memory problems, and greater life disruption, may have been more likely to seek medical help and receive a formal long COVID diagnosis, whereas those with milder symptoms may have been less inclined to consult a professional and therefore had higher memory scores in the self-reported group. Our study supports this hypothesis, as we found that the diagnosed long COVID group reported that they had more severe brain fog and that their symptoms had a greater impact on their life. A previous study has shown that more severe COVID-19 symptoms are linked to poorer cognitive function including memory deficits (Guo et al., 2022).

Several mechanisms have been proposed to explain the impact of long COVID on cognition including memory function. These include pathophysiological mechanisms that could impede brain function such as neuroinflammation, damage to neurons and astrocytes, vascular alterations, neurotropic effects, dysautonomia, hypoxia, blood coagulation, and metabolic changes (Bauer et al., 2022; Davis et al., 2023; Moller et al., 2023; Nalbandian et al., 2021; Natarajan et al., 2023; Plantone et al., 2024; Turner et al., 2023). In addition, long COVID could affect psychological wellbeing, leading to conditions such as anxiety, depression, post-traumatic stress disorder, or sleep disturbances which in turn could contribute to memory deficits (Aiyegbusi et al., 2021; Davis et al., 2023; Kennelly et al., 2023; Natarajan et al., 2023; Tedjasukmana et al., 2022).

A number of rehabilitation approaches have been proposed to treat long COVID cognitive symptoms (with mixed effectiveness) including cognitive training, electromagnetic brain stimulation, psychosocial, pharmaceutical, natural supplements, psychoeducation/compensatory skills training, telerehabilitation and physical rehabilitation such as hyperbaric oxygen therapy (Gorenshtein et al., 2024; Hawke et al., 2024; Moller et al., 2023; Salawu et al., 2020). A personalized clinical treatment plan that considers working memory deficits may help address both the cognitive as well as physical symptoms of long COVID.

### Study limitations

One limitation of our study is the use of a single online memory quiz that has not undergone formal psychometric validation against gold-standard neuropsychological assessments. While the quiz was designed based on established working memory paradigms and has previously demonstrated sensitivity to COVID-related cognitive differences (Baseler et al., 2022), we cannot make definitive claims about its reliability, validity, or specificity. However, our approach offers a pragmatic solution for screening larger populations for working memory difficulties in long COVID, which may then inform more detailed clinical evaluation. Our working memory quiz is currently being applied along with several standard neuropsychological tests including the Montreal Cognitive Assessment (MoCA) and the Modified Mini-Mental State Exam (3MSE) in a study evaluating cognitive function in patients with kidney disease undergoing hemodialysis (Aksoy et al., 2024). Future research should aim to validate this brief online measure against established clinical neuropsychological tests to determine its psychometric properties, including test–retest reliability, construct validity, discriminant validity, and sensitivity to change over time.

Prior to starting the memory quiz, the survey reminded participants of the importance of the study and to undertake the quiz in an environment without distractions. However, we acknowledge the inherent limitations of unsupervised online testing, including variable levels of distraction and compliance, which could affect performance of any participant regardless of long COVID status. Nonetheless, online testing offers significant advantages in terms of accessibility, particularly important for reaching individuals with mobility limitations including those with long COVID. The ecological validity of testing participants in more natural environments may, however, better reflect real-world cognitive functioning than laboratory-based assessments.

Our use of convenience sampling through social media, support groups, and professional networks may have introduced bias. For example, individuals engaged in long COVID support groups may be those who experience more persistent or severe symptoms, which could lead to an overestimation of cognitive difficulties. However, there may have also been an underrepresentation of those too unwell to complete the study, which could contribute to an underestimation of effects in the most affected group. The majority of our sample was based in the United Kingdom (83%), which may limit the generalizability of the findings worldwide. Capturing demographics and epidemiology of long COVID across countries is challenging because studies across the world often use different sampling methods, diagnostic criteria, and methodologies, making it difficult to consistently compare and combine datasets. However, the distribution of ages and gender in our sample are in line with the demographics published in a comprehensive review of long COVID which includes data from the Office of National Statistics in the United Kingdom (Greenhalgh et al., 2024).

Although an *a priori* power analysis was not conducted before data collection, with our final sample size of 1,064 participants (415 with long COVID and 649 controls) and an effect size of Hedges’ *g* = 0.552, the calculated statistical power was > 99%. Moreover, after correction for multiple comparisons, all statistically significant differences resulted in *p* < 0.005. Together, this indicates that our study is sufficiently powered to detect differences in working memory scores using our single rapid online test.

The purpose of the study was to compare working memory function in those with long COVID with those without long COVID, rather than to determine the specific contribution of all factors that may influence cognitive performance. Although several variables were significantly associated with working memory performance, the regression models explained only a modest proportion of the variance in memory scores (*R*^2^ = 0.114 and 0.079), indicating that additional factors not assessed in the present study are likely to contribute to individual differences in working memory function. We operated under the assumption that participants with pre-existing cognitive impairments or other conditions affecting working memory were equally distributed across the non-long COVID and long COVID cohorts, but this may not have been the case. Long COVID is a complex and heterogeneous condition that may disproportionately affect specific demographic and socioeconomic groups (Greenhalgh et al., 2024). Furthermore, fatigue, memory problems, sleep disturbance, anxiety, depression, and other co-occurring symptoms are common in individuals with long COVID and may contribute to variability in cognitive outcomes (Aiyegbusi et al., 2021; Davis et al., 2023; Kennelly et al., 2023; Nalbandian et al., 2021; Natarajan et al., 2023; O’Mahoney et al., 2023; Tedjasukmana et al., 2022). Educational attainment and socioeconomic status have also been associated with differences in cognitive and executive functioning, including memory and working memory performance (Migeot et al., 2022). As educational attainment, socioeconomic status, medication use, and pre-existing neurological or psychiatric conditions were not assessed in the present study, their potential contribution to the observed differences in working memory performance could not be evaluated. Consequently, residual confounding cannot be excluded, and the variables identified in this study should be interpreted as significant correlates of working memory performance rather than comprehensive determinants of cognitive outcomes in long COVID.

To maximize engagement, our memory quiz was intentionally designed to be relatively easy. Consequently, while the observed differences were significant, effect sizes were small-to-medium, yielding mean score differences of just 1–2 points out of a possible 16. Nevertheless, even modest reductions in working memory may affect everyday activities such as problem-solving, decision-making, reading comprehension, and maintaining conversations. Consistent with this, lower memory scores were associated with greater self-reported brain fog and a more severe impact of long COVID symptoms on daily life. While the cross-sectional design does not permit causal inference and the regression models explained only a modest proportion of the variance in memory scores, these findings suggest that working memory impairment may represent a clinically relevant component of the cognitive difficulties experienced by some individuals with long COVID. Consequently, the observed associations should be viewed as contributing to the growing evidence that working memory may be affected in long COVID, while recognizing that additional factors not assessed in the present study are also likely to influence cognitive outcomes. Our working memory quiz may have value as part of a broader assessment to help identify and characterize working memory difficulties in individuals with long COVID.

## Conclusion

Our results suggest that long COVID is associated with poorer working memory performance, particularly among participants reporting a clinical diagnosis of long COVID. Working memory performance was also associated with age, infection frequency, brain fog severity, and the perceived impact of symptoms on daily life. While these findings are consistent with cognitive difficulties reported in long COVID, they should be interpreted in the context of the study’s cross-sectional design, online assessment methodology, and the possibility that additional factors not measured in the present study may have influenced working memory performance. Nevertheless, our findings add to the growing body of evidence linking long COVID with objective cognitive impairment and support the use of rapid online assessments as a practical approach for large-scale evaluation of working memory difficulties in this population.

## Data Availability

The raw data supporting the conclusions of this article will be made available by the authors, without undue reservation.

## References

[ref1] Aghajani Mir M. (2023). Brain fog: a narrative review of the Most common mysterious cognitive disorder in COVID-19. Mol. Neurobiol. 61, 9915–9926. doi: 10.1007/s12035-023-03715-y, 37874482

[ref2] Aiyegbusi O. L. Hughes S. E. Turner G. Rivera S. C. McMullan C. Chandan J. S. . (2021). Symptoms, complications and management of long COVID: a review. J. R. Soc. Med. 114, 428–442. doi: 10.1177/01410768211032850, 34265229 PMC8450986

[ref3] Aksoy M. Hunter S. Asghar A. U. R. Bhandari S. (2024). A randomised controlled pilot trial protocol for patient led cognitive gamified training during haemodialysis. Sci. Rep. 14:30704. doi: 10.1038/s41598-024-79797-y, 39730448 PMC11680835

[ref4] Arbula S. Pisanu E. Bellavita G. Menichelli A. Lunardelli A. Furlanis G. . (2024). Insights into attention and memory difficulties in post-COVID syndrome using standardized neuropsychological tests and experimental cognitive tasks. Sci. Rep. 14:4405. doi: 10.1038/s41598-024-54613-9, 38388708 PMC10883994

[ref5] World Medical Association (2013). World medical association declaration of Helsinki: ethical principles for medical research involving human subjects. JAMA 310, 2191–2194. doi: 10.1001/jama.2013.281053 24141714

[ref6] Atchison C. J. Davies B. Cooper E. Lound A. Whitaker M. Hampshire A. . (2023). Long-term health impacts of COVID-19 among 242,712 adults in England. Nat. Commun. 14:6588. doi: 10.1038/s41467-023-41879-2, 37875536 PMC10598213

[ref7] Baddeley A. (2003). Working memory: looking back and looking forward. Nat. Rev. Neurosci. 4, 829–839. doi: 10.1038/nrn1201, 14523382

[ref8] Baddeley A. D. Hitch G. J. Allen R. J. (2019). From short-term store to multicomponent working memory: the role of the modal model. Mem. Cogn. 47, 575–588. doi: 10.3758/s13421-018-0878-5, 30478520

[ref9] Baseler H. A. Aksoy M. Salawu A. Green A. Asghar A. U. R. (2022). The negative impact of COVID-19 on working memory revealed using a rapid online quiz. PLoS One 17:e0269353. doi: 10.1371/journal.pone.0269353, 36374838 PMC9662713

[ref10] Bauer L. Laksono B. M. Vrij F. M. S. Kushner S. A. Harschnitz O. Riel D. (2022). The neuroinvasiveness, neurotropism, and neurovirulence of SARS-CoV-2. Trends Neurosci. 45, 358–368. doi: 10.1016/j.tins.2022.02.006, 35279295 PMC8890977

[ref11] Bertuccelli M. Ciringione L. Rubega M. Bisiacchi P. Masiero S. Del Felice A. (2022). Cognitive impairment in people with previous COVID-19 infection: a scoping review. Cortex 154, 212–230. doi: 10.1016/j.cortex.2022.06.002, 35780756 PMC9187867

[ref12] Blomberg B. Mohn K. G. Brokstad K. A. Zhou F. Linchausen D. W. Hansen B. A. . (2021). Long COVID in a prospective cohort of home-isolated patients. Nat. Med. 27, 1607–1613. doi: 10.1038/s41591-021-01433-3, 34163090 PMC8440190

[ref13] Bowe B. Xie Y. Al-Aly Z. (2022). Acute and postacute sequelae associated with SARS-CoV-2 reinfection. Nat. Med. 28, 2398–2405. doi: 10.1038/s41591-022-02051-3, 36357676 PMC9671810

[ref14] Brockmole J. R. Logie R. H. (2013). Age-related change in visual working memory: a study of 55,753 participants aged 8-75. Front. Psychol. 4:12. doi: 10.3389/fpsyg.2013.00012, 23372556 PMC3557412

[ref15] Bungenberg J. Humkamp K. Hohenfeld C. Rust M. I. Ermis U. Dreher M. . (2022). Long COVID-19: objectifying most self-reported neurological symptoms. Ann. Clin. Transl. Neurol. 9, 141–154. doi: 10.1002/acn3.51496, 35060361 PMC8862437

[ref16] Chai W. J. Abd Hamid A. I. Abdullah J. M. (2018). Working memory from the psychological and neurosciences perspectives: a review. Front. Psychol. 9:401. doi: 10.3389/fpsyg.2018.00401, 29636715 PMC5881171

[ref17] Cheetham N. J. Penfold R. Giunchiglia V. Bowyer V. Sudre C. H. Canas L. S. . (2023). The effects of COVID-19 on cognitive performance in a community-based cohort: a COVID symptom study biobank prospective cohort study. EClinicalMedicine 62:102086. doi: 10.1016/j.eclinm.2023.102086 37654669 PMC10466229

[ref18] Corbett A. Williams G. Creese B. Hampshire A. Hayman V. Palmer A. . (2023). Cognitive decline in older adults in the UK during and after the COVID-19 pandemic: a longitudinal analysis of PROTECT study data. Lancet Healthy Longev. 4, e591–e599. doi: 10.1016/S2666-7568(23)00187-3, 37924840 PMC10720396

[ref19] Cui R. Gao B. Ge R. Li M. Li M. Lu X. . (2024). The effects of COVID-19 infection on working memory: a systematic review. Curr. Med. Res. Opin. 40, 217–227. doi: 10.1080/03007995.2023.2286312, 38008952

[ref20] Davis H. E. McCorkell L. Vogel J. M. Topol E. J. (2023). Long COVID: major findings, mechanisms and recommendations. Nat. Rev. Microbiol. 21, 133–146. doi: 10.1038/s41579-022-00846-2, 36639608 PMC9839201

[ref21] Diamond A. (2013). Executive functions. Annu. Rev. Psychol. 64, 135–168. doi: 10.1146/annurev-psych-113011-143750, 23020641 PMC4084861

[ref22] Diexer S. Klee B. Gottschick C. Xu C. Broda A. Purschke O. . (2023). Association between virus variants, vaccination, previous infections, and post-COVID-19 risk. Int. J. Infect. Dis. 136, 14–21. doi: 10.1016/j.ijid.2023.08.019, 37634619

[ref23] Elliott P. Bodinier B. Eales O. Wang H. Haw D. Elliott J. . (2022). Rapid increase in omicron infections in England during December 2021: REACT-1 study. Science 375, 1406–1411. doi: 10.1126/science.abn8347, 35133177 PMC8939772

[ref24] Feltz-Cornelis C. Turk F. Sweetman J. Khunti K. Gabbay M. Shepherd J. . (2024). Prevalence of mental health conditions and brain fog in people with long COVID: a systematic review and meta-analysis. Gen. Hosp. Psychiatry 88, 10–22. doi: 10.1016/j.genhosppsych.2024.02.009, 38447388

[ref25] Finamore P. Arena E. Lupoi D. Savito L. Nunzio F. Furbatto M. . (2024). Long COVID syndrome: a. narrative review on burden of age and vaccination. J. Clin. Med. 13:4756. doi: 10.3390/jcm13164756 39200898 PMC11355827

[ref26] Finterer J. Scorza F. A. (2022). A retrospective analysis of clinically confirmed long post-COVID vaccination syndrome. J Clin Transl Res 8, 506–508. doi: 10.18053/jctres.08.202206.008, 36452006 PMC9706319

[ref27] Gao Z. Xu Y. Sun C. Wang X. Guo Y. Qiu S. . (2021). A systematic review of asymptomatic infections with COVID-19. J. Microbiol. Immunol. Infect. 54, 12–16. doi: 10.1016/j.jmii.2020.05.001, 32425996 PMC7227597

[ref28] Gorenshtein A. Liba T. Leibovitch L. Stern S. Stern Y. (2024). Intervention modalities for brain fog caused by long-COVID: systematic review of the literature. Neurol. Sci. 45, 2951–2968. doi: 10.1007/s10072-024-07566-w, 38695969 PMC11176231

[ref29] Graham E. L. Clark J. R. Orban Z. S. Lim P. H. Szymanski A. L. Taylor C. . (2021). Persistent neurologic symptoms and cognitive dysfunction in non-hospitalized Covid-19 ‘long haulers’. Ann. Clin. Transl. Neurol. 8, 1073–1085. doi: 10.1002/acn3.51350, 33755344 PMC8108421

[ref30] Greenhalgh T. Sivan M. Perlowski A. Nikolich J. Z. (2024). Long COVID: a clinical update. Lancet 404, 707–724. doi: 10.1016/S0140-6736(24)01136-X, 39096925

[ref31] Guo P. Benito Ballesteros A. Yeung S. P. Liu R. Saha A. Curtis L. . (2022). COVCOG 2: cognitive and memory deficits in long COVID: a second publication from the COVID and cognition study. Front. Aging Neurosci. 14:804937. doi: 10.3389/fnagi.2022.804937, 35370620 PMC8967943

[ref32] Hampshire A. Azor A. Atchison C. Trender W. Hellyer P. J. Giunchiglia V. . (2024). Cognition and memory after Covid-19 in a large community sample. N. Engl. J. Med. 390, 806–818. doi: 10.1056/NEJMoa2311330, 38416429 PMC7615803

[ref33] Hawke L. D. Nguyen A. T. P. Wang W. Brown E. E. Xu D. Deuville S. . (2024). Systematic review of interventions for mental health, cognition and psychological well-being in long COVID. BMJ Ment. Health 27, 1–11. doi: 10.1136/bmjment-2024-301133, 39384321 PMC11474695

[ref34] Jennings G. Monaghan A. Xue F. Duggan E. Romero-Ortuno R. (2022). Comprehensive clinical characterisation of brain fog in adults reporting long COVID symptoms. J. Clin. Med. 11:3440. doi: 10.3390/jcm11123440, 35743516 PMC9224578

[ref35] Kalak G. Jarjou’i A. Bohadana A. Wild P. Rokach A. Amiad N. . (2022). Prevalence and persistence of symptoms in adult COVID-19 survivors 3 and 18 months after discharge from hospital or corona hotels. J. Clin. Med. 11:7413. doi: 10.3390/jcm11247413, 36556030 PMC9784691

[ref36] Katsuki F. (2012). Unique and shared roles of the posterior parietal and dorsolateral prefrontal cortex in cognitive functions. Front. Integr. Neurosci. 6:17. doi: 10.3389/fnint.2012.00017, 22563310 PMC3342558

[ref37] Kennelly C. E. Nguyen A. T. P. Sheikhan N. Y. Strudwick G. Ski C. F. Thompson D. R. . (2023). The lived experience of long COVID: a qualitative study of mental health, quality of life, and coping. PLoS One 18:e0292630. doi: 10.1371/journal.pone.0292630, 37831706 PMC10575511

[ref38] Kim Y. Bae S. Chang H. H. Kim S. W. (2023). Long COVID prevalence and impact on quality of life 2 years after acute COVID-19. Sci. Rep. 13:11207. doi: 10.1038/s41598-023-36995-4, 37433819 PMC10336045

[ref39] Lam G. Y. Damant R. W. Ferrara G. Lim R. K. Stickland M. K. Ogando N. S. . (2023). Characterizing long-COVID brain fog: a retrospective cohort study. J. Neurol. 270, 4640–4646. doi: 10.1007/s00415-023-11913-w, 37555926

[ref40] Lauria A. Carfi A. Benvenuto F. Bramato G. Ciciarello F. Rocchi S. . (2023). Neuropsychological measures of post-COVID-19 cognitive status. Front. Psychol. 14:1136667. doi: 10.3389/fpsyg.2023.1136667 37492442 PMC10363721

[ref41] Migeot J. Calivar M. Granchetti H. Ibáñez A. Fittipaldi S. (2022). Socioeconomic status impacts cognitive and socioemotional processes in healthy ageing. Sci. Rep. 12:6048. doi: 10.1038/s41598-022-09580-4, 35410333 PMC9001669

[ref42] Millet C. Narvaneni S. Roman S. Horani G. Chaudhry S. Michael P. . (2022). Symptoms persist in patients two years after COVID-19 infection: a prospective follow-up study. Clin. Microbiol. Infect. 28, 1505–1507. doi: 10.1016/j.cmi.2022.06.008, 35724867 PMC9212679

[ref43] Miskowiak K. W. Johnsen S. Sattler S. M. Nielsen S. Kunalan K. Rungby J. . (2021). Cognitive impairments four months after COVID-19 hospital discharge: pattern, severity and association with illness variables. Eur. Neuropsychopharmacol. 46, 39–48. doi: 10.1016/j.euroneuro.2021.03.019, 33823427 PMC8006192

[ref44] Miskowiak K. W. Pedersen J. K. Gunnarsson D. V. Roikjer T. K. Podlekareva D. Hansen H. . (2023). Cognitive impairments among patients in a long-COVID clinic: prevalence, pattern and relation to illness severity, work function and quality of life. J. Affect. Disord. 324, 162–169. doi: 10.1016/j.jad.2022.12.122, 36586593 PMC9795797

[ref45] Moller M. Borg K. Janson C. Lerm M. Normark J. Niward K. (2023). Cognitive dysfunction in post-COVID-19 condition: mechanisms, management, and rehabilitation. J. Intern. Med. 294, 563–581. doi: 10.1111/joim.13720, 37766515

[ref46] Nalbandian A. Sehgal K. Gupta A. Madhavan M. V. McGroder C. Stevens J. S. . (2021). Post-acute COVID-19 syndrome. Nat. Med. 27, 601–615. doi: 10.1038/s41591-021-01283-z 33753937 PMC8893149

[ref47] Natarajan A. Shetty A. Delanerolle G. Zeng Y. Zhang Y. Raymont V. . (2023). A systematic review and meta-analysis of long COVID symptoms. Syst. Rev. 12:88. doi: 10.1186/s13643-023-02250-0, 37245047 PMC10220332

[ref48] NHS. (2021). Post-COVID Syndrome Long COVID National Health Service England. Available online at: https://www.england.nhs.uk/coronavirus/post-covid-syndrome-long-covid/ (Accessed May 13, 2026).

[ref49] NICE. (2020). COVID-19 Rapid Guideline: Managing the Long-Term Effects of COVID-19 National Institute for Health and Care Excellence. Available online at: https://www.nice.org.uk/guidance/ng188/chapter/1-Identification (Accessed May 13, 2026). 33555768

[ref50] Nordvig A. S. Rajan M. Lau J. D. Kingery J. R. Mahmud M. Chiang G. C. . (2023). Brain fog in long COVID limits function and health status, independently of hospital severity and preexisting conditions. Front. Neurol. 14:1150096. doi: 10.3389/fneur.2023.1150096 37251229 PMC10213727

[ref51] O’Mahoney L. L. Routen A. Gillies C. Ekezie W. Welford A. Zhang A. . (2023). The prevalence and long-term health effects of long covid among hospitalised and non-hospitalised populations: a systematic review and meta-analysis. EClinicalMedicine 55:101762. doi: 10.1016/j.eclinm.2022.101762 36474804 PMC9714474

[ref52] Office for National Statistics. (2023). Coronavirus (COVID-19) Latest Insights: Infections. Newport: Office for National Statistics. Available online at: https://www.ons.gov.uk/peoplepopulationandcommunity/healthandsocialcare/conditionsanddiseases/articles/coronaviruscovid19latestinsights/infections. (Accessed May 13, 2026).

[ref53] Park D. C. Lautenschlager G. Hedden T. Davidson N. S. Smith A. D. Smith P. K. (2002). Models of visuospatial and verbal memory across the adult life span. Psychol. Aging 17, 299–320. doi: 10.1037/0882-7974.17.2.299, 12061414

[ref54] Plantone D. Stufano A. Righi D. Locci S. Iavicoli I. Lovreglio P. . (2024). Neurofilament light chain and glial fibrillary acid protein levels are elevated in post-mild COVID-19 or asymptomatic SARS-CoV-2 cases. Sci. Rep. 14:6429. doi: 10.1038/s41598-024-57093-z 38499607 PMC10948776

[ref55] Premraj L. Kannapadi N. V. Briggs J. Seal S. M. Battaglini D. Fanning J. . (2022). Mid and long-term neurological and neuropsychiatric manifestations of post-COVID-19 syndrome: a meta-analysis. J. Neurol. Sci. 434:120162. doi: 10.1016/j.jns.2022.120162, 35121209 PMC8798975

[ref56] Repovs G. Baddeley A. (2006). The multi-component model of working memory: explorations in experimental cognitive psychology. Neuroscience 139, 5–21. doi: 10.1016/j.neuroscience.2005.12.061, 16517088

[ref57] Salawu A. Green A. Crooks M. G. Brixey N. Ross D. H. Sivan M. (2020). A proposal for multidisciplinary tele-rehabilitation in the assessment and rehabilitation of COVID-19 survivors. Int. J. Environ. Res. Public Health 17. doi: 10.3390/ijerph17134890, 32645876 PMC7369849

[ref58] Schroeder R. W. Twumasi-Ankrah P. Baade L. E. Marshall P. S. (2012). Reliable digit span: a systematic review and cross-validation study. Assessment 19, 21–30. doi: 10.1177/1073191111428764, 22156721

[ref59] Stavem K. Einvik G. Tholin B. Ghanima W. Hessen E. Lundqvist C. (2022). Cognitive function in non-hospitalized patients 8-13 months after acute COVID-19 infection: a cohort study in Norway. PLoS One 17:0273352. doi: 10.1371/journal.pone.0273352, 35994448 PMC9394790

[ref60] Tanabe A. Osaka N. (2009). Picture span test: measuring visual working memory capacity involved in remembering and comprehension. Behav. Res. Methods 41, 309–317. doi: 10.3758/BRM.41.2.309, 19363171

[ref61] Tedjasukmana R. Budikayanti A. Islamiyah W. R. Witjaksono A. Hakim M. (2022). Sleep disturbance in post COVID-19 conditions: prevalence and quality of life. Front. Neurol. 13:1095606. doi: 10.3389/fneur.2022.1095606, 36698905 PMC9869804

[ref62] Thronicke A. Hinse M. Weinert S. Jakubowski A. Grieb G. Matthes H. (2022). Factors associated with self-reported post/long-COVID-A real-world data study. Int. J. Environ. Res. Public Health 19:16124. doi: 10.3390/ijerph192316124, 36498197 PMC9738553

[ref63] Turner S. Khan M. A. Putrino D. Woodcock A. Kell D. B. Pretorius E. (2023). Long COVID: pathophysiological factors and abnormalities of coagulation. Trends Endocrinol. Metab. 34, 321–344. doi: 10.1016/j.tem.2023.03.002, 37080828 PMC10113134

[ref64] WHO. (2022). Post COVID-19 Condition Long COVID World Health Organization. Available online at: https://www.who.int/europe/news-room/fact-sheets/item/post-covid-19-condition (Accessed May 13, 2026).

[ref65] Zawilska J. B. Kuczynska K. (2022). Psychiatric and neurological complications of long COVID. J. Psychiatr. Res. 156, 349–360. doi: 10.1016/j.jpsychires.2022.10.045, 36326545 PMC9582925

[ref66] Zhao S. Toniolo S. Hampshire A. Husain M. (2023). Effects of COVID-19 on cognition and brain health. Trends Cogn. Sci. 27, 1053–1067. doi: 10.1016/j.tics.2023.08.008, 37657964 PMC10789620

[ref67] Ziauddeen N. Gurdasani D. O’Hara M. E. Hastie C. Roderick P. Yao G. . (2022). Characteristics and impact of long covid: findings from an online survey. PLoS One 17:e0264331. doi: 10.1371/journal.pone.0264331, 35259179 PMC8903286

